# Increased Cerebral Vein Diameters Are Associated with Age and White Matter Hyperintensity

**DOI:** 10.3390/biomedicines14020477

**Published:** 2026-02-21

**Authors:** Gokhan Duygulu, Fulya Kahraman

**Affiliations:** Department of Radiology, Atatürk Training and Research Hospital, Izmir 35150, Türkiye; fulyakahraman@msn.com

**Keywords:** white matter hyperintensity, Fazekas, internal cerebral veins, thalamostriate veins, anterior septal veins

## Abstract

**Objective:** White matter hyperintensity (WMH) is one of the most common and prominent changes seen in elderly individuals, especially on MRI. WMH is associated with serious conditions such as hemorrhagic and ischemic stroke, depression and dementia. Recently, the relationship between cerebral venous diameter and WMH was described. This study aimed to investigate the relationship between the Fazekas scale, which evaluates the severity of WMH, and cerebral vein diameters, age and clinical outcomes analysis. **Materials and Methods:** MRI images of 660 patients were examined retrospectively. FLAIR and SWI (MiniP) images were used to evaluate WMH and cerebral vein diameters. Internal cerebral veins (ICV), thalamostriate veins (TSV), anterior septal veins (ASV) and superior sagittal sinus (SSS) diameters were measured. Cerebral vein diameters were compared with age, WMH, hypertension, hyperlipidemia, diabetes mellitus, lacunar infarct and microhemorrhage presence. **Results**: In the presence of hypertension, hyperlipidemia, diabetes, lacunar infarction and microhemorrhage, Fazekas score, mean ICV-right, ICV-left, ASV-right, ASV-left, TSV-right and TSV-left values were significantly higher. The mean ICV-right, ICV-left, ASV-right, ASV-left, TSV-right and TSV-left values of the middle-aged and elderly groups were significantly higher than the young group. A strong positive correlation was observed between age and mean ICV-right, ICV-left, ASV-right and ASV-left values, while a moderate positive correlation was shown with TSV-right and TSV-left values. A weak negative correlation was determined with SSS values. **Conclusions:** Cerebral vein diameter increases with age and is associated with the severity of WMH. Clinicians can monitor cerebral vein diameter to predict the severity of WMH.

## 1. Introduction

White matter hyperintensities (WMHs) are common radiological findings in the brains of older people on T2-weighted fluid-attenuated inversion imaging (FLAIR) [1]. WMHs, thought to represent microvascular lesions in the brain, have been suggested to be associated with cognitive decline, microstructural damage, and brain atrophy [2]. WMHs are also considered an important indicator of cerebral small vessel disease (CSVD) [3]. The prevalence and severity of WMHs are age-dependent, and they are regarded as one of the most common imaging indicators of brain aging. Even though they can be preliminarily clinically silent, WMHs cannot help but be linked to the further degradation of the cognitive state, impaired mobility, emotional disturbances, and the high risk of stroke and dementia. Thus, clinically, the proper evaluation of the burden of WMH is significant to risk stratification and the interpretation of underlying cerebrovascular pathways [4,5].

The etiological factors and physiopathology of WMHs have not been fully elucidated. Some risk factors have been identified such as diabetes mellitus, hypertension and age. Especially in elderly and/or hypertensive individuals, the incidence of WMH is higher and the rate of progression of the disease is higher [6]. Hypoperfusion of the white matter has been implicated for the pathophysiology of WMH [7]. Studies have shown that a decrease in cerebral perfusion is associated with the severity of WMH and cognitive impairment [8,9]. Moreover, regional hypoperfusion results from cerebral small vessel lesions and hypertension has been shown to be an important risk factor for these vascular lesions [10].

Many radiological quantitative and qualitative scales characterize WMH. In recent studies, the relationship between WMH and cerebral vein diameters in elderly individuals has been demonstrated [10]. Visual rating scales are the most popular methods of WMH evaluation that are applied in clinical and research practices because they are quite convenient. The Fazekas scale [11] is a widely used visual rating method to rate WMHs in both research and clinical practice. The Fazekas scale is a histopathologically validated and easily applicable method that allows qualitative and quantitative analysis of WMH [11]. Fazekas scale is a semi-qualitative visual scale of assessment of WMHs in FLAIR MRI, which categorizes them in four levels ranging between absence of lesions and large confluent hyperintensities [12,13]. It has shown good inter-rater reliability and histopathological validity and it seems to be best applied to retrospective studies in which automated volumetric analysis may be unavailable. This study aimed to investigate the relationship between the Fazekas scale, which evaluates the severity of WMH, and cerebral vein diameters, with age and clinical outcomes analysis. There is growing evidence that changes in the structure of the cerebral veins, such as changes in vein size, may be involved in the impaired drainage and hypoperfusion, and thus the development of WMHs.

## 2. Materials and Methods

### 2.1. Study Subjects

In this study, brain MRIs taken between January 2023 and May 2023 at Izmir Katip Çelebi, Atatürk Training and Research Hospital, department of radiology, were retrospectively examined. Brain MRI examinations acquired between the following timeline were retrospectively analyzed in this study. Approval for the study was received from Izmir Katip Çelebi University Faculty of Medicine clinical ethics committee (Application no: 2023-GOKAE-0260, date: 18 May 2023, decision no: 0236).

Exclusion criteria for the study were determined as having dementia, having pathologies such as brain tumor, metastasis, intoxication, infection that may cause white matter disease, having a head injury, having hemorrhage, having a history of surgery, radiotherapy or chemotherapy and having motion or susceptibility artifacts in MR image quality. These exclusion criteria were applied to minimize potential confounding factors that could influence white matter pathology or cerebral venous measurements.

### 2.2. MRI Examination and Vein Measurements

All patients were examined with a 1.5T MRI system (Siemens Magnetom Aera, Siemens Healthcare, Erlangen, Germany) using a 16-channel brain phased array coil. Brain MRIs were performed with transverse T1-weighted imaging (T1W), T2-weighted imaging (T2W), and T2-weighted FLAIR and SWI. Sequence parameters used in the study: T1W (TR = 450 ms, TE = 8.9 ms, flip angle = 90°, FOV = 230 × 185 × 140mm, section thickness = 5.0 mm), T2W FLAIR (TR = 8000 ms, TE = 86 ms, inversion recovery time = 2372 ms, flipangle = 150°, FOV = 230 × 230 × 183 mm, section thickness = 5.0 mm), and SWI (TR = 49 ms, TE = 40 ms, flip angle = 15°, FOV = 230 × 180 × 130 mm, section thickness = 1.0 mm) sequences.

FLAIR and SWI (MiniP) images of the patients were used to evaluate WMHs and cerebral vein diameters. Internal cerebral veins (ICV), anterior septal veins (ASV), thalamostriate veins (TSV) and superior sagittal sinuses (SSS) were visually identified on transverse SWI (MinIP) images. Diameters of cerebral vessels were measured and recorded as previously described [14]. The images were also examined for the presence of microhemorrhage and lacunar infarct. MR images were evaluated by two experienced radiologists. All MRI measurements and WMH assessments were independently performed by two experienced radiologists, and inter-rater reliability was evaluated using intraclass correlation coefficients.

### 2.3. Examination of WMHs

**Figure 1:**
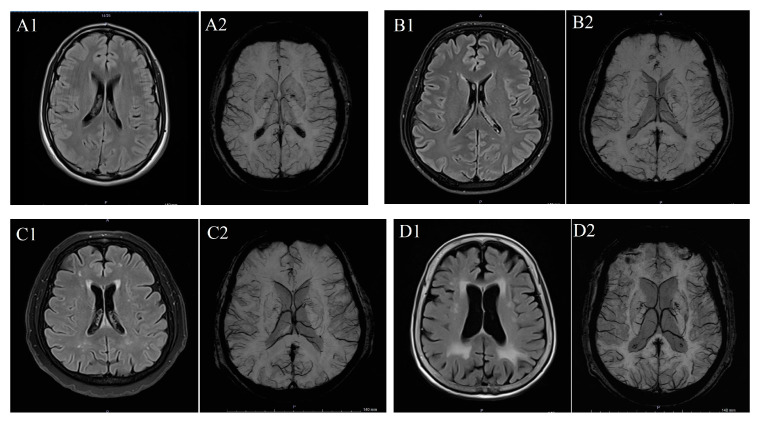
(**A**) T2 FLAIR (**A1**) and SW (**A2**) imaging in a patient with Fazekas 0 case, (**B**) T2 FLAIR (**B1**) and SW (**B2**) imaging in a patient with Fazekas 1 case, (**C**) T2 FLAIR (**C1**) and SW (**C2**) imaging iin a patient with Fazekas 2 case and (**D**) T2 FLAIR (**D1**) and SW (**D2**) imaging in a patient with Fazekas 3 case.

To determine WMH severity, the Fazekas scale was applied to FLAIR MRI data in the axial plane, following standard guidelines [11]. According to this scale, WMH was scored in categories between 0 and 3: “0” meaning absence of WMH; “1” meaning dotted WMH; “2” meaning early combined WMH; and “3” meaning WMH in large combined areas (Figure 1) [15]. The scale was selected due to its widespread clinical use, reproducibility, and suitability for retrospective MRI-based evaluation of WMHs.

### 2.4. Statistical Analysis

Statistical evaluation was made using SPSS version 20.0 for Windows statistical software. The Kolmogorov–Smirnov test was used to evaluate the suitability of the measured data for normal distribution. Mean, standard error, minimum and maximum values of continuous variables, and n and percentage values of categorical variables were given. The Chi-Square test was performed to compare categorical data and the Kruskal–Wallis test was performed to compare continuous data. The Mann–Whitney test was used for pairwise comparisons. The Spearman correlation analysis was performed for the correlation between data. For statistical analysis results, a *p*-value of less than 0.05 was considered significant. Non-parametric statistical tests were preferred due to the ordinal nature of the Fazekas scale and the non-normal distribution of several continuous variables.

## 3. Results

Diameter measurements of cerebral veins and Fazekas scoring had ICC values indicating good inter-rater reliability (ICC_Fazekas_ = 0.98 [95% confidence interval (CI), 0.98–0.99], ICC_SSS_ = 0.99 [95% CI, 0.99–0.99], ICC_ICV_ = 0.99 [95% CI, 0.99–0.99], ICC_ASV_ = 0.91 [95% CI, 0.89–0.92] and ICC_TSV_ = 0.83 [95% CI, 0.81–0.85]).

Descriptive statistical results of the participants are given in Table 1. Of the 660 participants, 413 (67.1%) were female and 247 (32.9%) were male, and the mean age of the participants was 53.47 ± 0.678. Two-hundred-and-seventeen (32.9%) individuals had hypertension, 135 (20.5%) had hyperlipidemia, and 134 (20.3%) had diabetes mellitus. According to Fazekas score, 142 (21.5%) of the participants were stage 0, 384 (58.2%) were stage 1, 74 (11.2%) were stage 2 and 60 (9.1%) were stage 3.

Comparison of participants’ data by gender is given in Table 2. The presence of hypertension was significantly higher in female participants than in male participants (*p* = 0.006). The mean ICV-right (*p* = 0.021), ICV-left (*p* = 0.011) and SSS (*p* = 0.001) values of male participants were significantly higher than female participants. Other parameters did not differ by gender. It was determined that the mean ICV-right, ICV-left, ASV-right, ASV-left, TSV-right and TSV-left values of individuals with hypertension, hyperlipidemia and diabetes mellitus were significantly higher and the mean SSS values were significantly lower than those of individuals without hypertension, hyperlipidemia and diabetes mellitus. The mean ICV-right, ICV-left, ASV-right, ASV-left, TSV-right and TSV-left values of individuals with lacunar infarcts were significantly higher than those of participants without lacunar infarcts. The mean ICV-right, ICV-left, ASV-right, ASV-left and TSV-right values of the participants with microhemorrhage were significantly higher and the mean SSS values were significantly lower than the participants without microhemorrhage (Table 3).

As the Fazekas score increased, the mean ICV-right, ICV-left, ASV-right, ASV-left, TSV-right and TSV-left values increased significantly. As the Fazekas score increased, the mean SSS value decreased, and stage 2 was significantly lower than stage 0, and stage 3 was significantly lower than stage 0 and stage 1 (Table 4). Additionally, it was observed that the Fazekas score increased significantly as age increased and in the presence of hypertension, hyperlipidemia, diabetes mellitus, lacunar infarction and microhemorrhage (Table 4).

Individuals less than 40 years of age were grouped as youth, individuals 40–60 years of age were grouped as middle-aged, and individuals over 60 years of age were grouped as elderly. The mean ICV-right, ICV-left, ASV-right, ASV-left, TSV-right and TSV-left values of the middle-aged and elderly groups were significantly higher than the young group (Table 5). The mean ICV-right, ICV-left, ASV-right, ASV-left, TSV-right and TSV-left values of the elderly group were significantly higher than the middle-aged group. The mean SSS values of the middle-aged and elderly groups were significantly lower than the young group. The mean SSS values of the elderly group were significantly lower than the young group. A strong positive correlation was observed between age and mean ICV-right, ICV-left, ASV-right and ASV-left values, and a moderate positive correlation with TSV-right and TSV-left. A weak negative correlation was shown with SSS values. When the presence of hypertension, hyperlipidemia, diabetes mellitus, lacunar infract and microhemorrhage was adjusted, there was a strong positive correlation between age and mean ICV-right and ASV-right values, and a moderate positive correlation was observed between age and mean IVC-left, ASV-left, TSV-right and TSV-left values (Table 6).

The Kolmogorov–Smirnov test showed that all continuous variables (age, Fazekas score, bilateral ICV, ASV, TSV, and SSS) significantly deviated from normal distribution (all *p* < 0.001). Therefore, non-parametric statistical methods were applied for subsequent analyses (Table 7). Spearman analysis demonstrated strong positive correlations between Fazekas score and bilateral ICV (r = 0.71 and 0.69) and ASV (r = 0.67 and 0.65), with moderate correlations for bilateral TSV (r = 0.48 and 0.46); all remained significant after adjustment (adjusted *p* < 0.001). Although SSS showed a weak negative correlation (r = −0.21), this did not remain significant after correction (adjusted *p* = 0.063). These findings indicate robust associations between venous parameters and WMH severity (Table 8).

Quade’s test revealed significant adjusted group differences for bilateral ICV, ASV, and TSV (all *p* < 0.001). The largest effect sizes were observed for ICV (partial η^2^ ≈ 0.17–0.18), followed by ASV (≈0.14–0.15) and TSV (≈0.07–0.08). SSS was not significant (*p* = 0.091; partial η^2^ = 0.01). This suggests that venous measurements remain independently associated with WMH severity after covariate adjustment, particularly ICV (Table 9).

Random Forest modeling identified age as the strongest predictor of WMH severity (100% relative importance). Hypertension, diabetes, and hyperlipidemia were also substantial contributors (30–47%). Venous parameters, particularly bilateral ICV (25–26%), showed meaningful independent importance within the model (Table 10). SHAP analysis demonstrated a progressive, non-linear increase in WMH contribution with advancing age. Age < 40 years showed a protective effect, whereas contributions increased from mild (40–60 years) to moderate (61–80 years) and became strongest in those >80 years (Table 11).

## 4. Discussion

In this retrospective study, the relationship of WMH and cerebral vein diameters with age and some clinical risk factors was evaluated. The presence of hypertension, hyperlipidemia and diabetes mellitus caused an increase in both cerebral vein diameters and WMH severity. It was determined that cerebral vein diameters and WMH severity increased with age, and the increase in cerebral vein diameters was strongly positively correlated with age. WMHs are known to be influenced by a wide range of factors beyond those evaluated in the present study, including socioeconomic status, lifestyle factors, inflammatory markers, genetic susceptibility, and psychological variables. Due to the retrospective nature of this analysis, such variables were not available and therefore could not be included. This limitation should be considered when interpreting the observed associations.

WMH, which is one of the most common and prominent changes seen especially in MRI of elderly individuals, is associated with serious conditions such as hemorrhagic and ischemic stroke, depression and dementia [2]. As a result of the studies conducted, researchers stated that WMH may occur as an inevitable consequence of aging. However, due to the widespread negative effects of WMH, it is very important to reveal the factors that cause WMH in order to both delay the age of onset and slow down its progression. Hypoperfusion due to cerebrovascular autoregulation disorder, blood–brain barrier disorder, and axonal loss and demyelination due to ischemia and hypoxia are responsible for the pathophysiology of WMH [16,17]. At the same time, cardiovascular risk factors such as hypertension, dyslipoproteinemia, atherosclerosis, diabetes mellitus, poor lifestyle and age have been identified as risk factors for WMH [18,19].

Although various risk factors related to arterial pathology have been identified for WMH, there are not enough studies on changes in cerebral vessels. Parenchymal damage caused by CSVD manifests itself as WMH, microhemorrhages, lacunas and enlarged perivascular spaces (PVSs) [20,21]. PVHs play a role in the removal of metabolic byproducts. There is a relationship between enlarged PVH and enlarged cerebral vein diameter [22]. It has been shown that there is a relationship between venous collagen deposition and WMHs in brain tissues examined after death [23]. Another study documented higher WMH volume and higher PVH volume with deep medullary vein damage [24]. As a matter of fact, Houck and colleagues demonstrated that there is a correlation between WMH volume and some vessel diameters in the brain, such as the SSS, ICV and Rosenthal basal vessels [25]. Huang et al. [14] documented that cerebral vein diameters increase as the severity of WMH increases. Researchers have observed that ICV and TCV diameters increase in middle-aged and elderly people, especially as the severity of WMH increases. Since SWI is highly sensitive to blood oxygenation differences between venous blood and brain tissue, it was used to measure cerebral vein diameter in this study [26,27]. The relationship between WMH and cerebral diameters is known. However, to our knowledge, there is no study investigating the relationship between Fazekas score and cerebral vein diameters. The fact that the number of patients in our study is higher than previous studies and that it is the first study investigating the relationship between Fazekas score and vein diameters makes our study unique.

We determined that as the Fazekas score increased, ICV, ASV and TCV diameters increased, while CNS diameter decreased. According to these results of our study, we can have a rough idea about cerebral vein diameters just by performing Fazekas scoring on MRI. These results of our study confirm the results of previous studies showing a relationship between cerebral vein diameter and WMH severity. In the light of the above studies, we suggest that the increase in diameter in the cerebral veins may be caused by collagen accumulation in the walls of the veins, which may cause narrowing of the lumen of the cerebral veins, resulting in ischemic WMH.

Hypertension, hyperlipidemia, and diabetes mellitus are risk factors associated with WMHs. Hypertension is linked to the pathogenesis of WMH due to disruption of systemic hemodynamics, endothelial dysfunction, and the development of vascular stiffness [6,28]. Moreover, studies have shown a causal relationship between hypertension and cerebrovascular venous wall sclerosis and thickening due to collagenosis [29,30]. Similarly, people with diabetes have been documented to have more and larger WMHs than non-diabetic people [31,32,33]. Del Bene et al. [34] emphasized that there is a close relationship between type 2 diabetes and the presence and severity of WMHs. Type 2 diabetes has been suggested to be an independent risk factor for WMHs [35]. In our study, it was determined that the number of individuals with Fazekas scores of 2 and 3 in those with hypertension, hyperlipidemia or diabetes mellitus was higher than in those without hypertension, hyperlipidemia or diabetes mellitus. The results of our study confirm the results of previous studies showing that hypertension, hyperlipidemia or diabetes mellitus increase the severity of WMH.

It is known that WMH is more common in older individuals and its severity increases with age. In their study with 682 older adults, Houck et al. [25] found a statistically significant relationship between ICV diameter and WMH severity. However, they did not group the patients according to age. In their study, Huang et al. [14] documented that the rate of WMH was quite low in young people and that patients with mild WMH were the majority in middle-aged and elderly patients. They also determined that the number of patients with moderate and severe WMH increased with increasing age. Huang et al. [14] also examined the change in cerebral vein diameters with age. They reported that there is a tendency for the diameters of TSV and ICV to increase with age, and the strongest relationship between age and vein diameters is the increase in ICV diameter. In our study, the lowest vein diameters were determined in young people and the highest vein diameters in the elderly. A significant difference was observed between the groups in terms of ICV, ASV, and TSV diameters.

Moreover, there was a strong positive correlation between age and ICV and ASV diameter, while a moderate positive correlation between TSV diameter. Apart from age, it is known that some risk factors such as hypertension, hyperlipidemia and diabetes mellitus cause both an increase in cerebral vein diameter and an increase in the severity of WMH. Although studies by Huang et al. showed that cerebral vein diameters and WMH severity increased with age, the effect of other risk factors was not evaluated. In our study, it was determined that age, as well as hypertension, hyperlipidemia and diabetes mellitus, caused an increase in both cerebral vein diameters and WMH severity. After adjustment for hypertension, hyperlipidemia, diabetes mellitus, lacunar infarction and microhemorrhage, a new correlation was made between age and cerebral vein diameters. A strong correlation was determined between adjusted age and ICV-right and ASV-right diameters, and a moderate correlation was determined between adjusted age and other vein diameters.

### Limitations

This research has got a number of limitations that can be noted. First, the nature of the study, which is single-center and retrospective, limited the analysis to the already collected variables and did not allow the creation of new data and the re-definition of existing ones. Second, WMHs are impacted by a vast diversity of factors such as socioeconomic status, lifestyle, inflammatory and genetic, and psychological variables, but such variables were not included in the current dataset and as such, could not be investigated. Third, age was not measured as a continuous variable, but rather in deterministic categories, which could have restricted the specificity of the age-related differences on WMH severity and cerebral vein diameters. Moreover, they did not conduct state-of-the-art methods of analysis such as machine-learning-based models or feature-level image analyses, since these methods need to have access to raw imaging data and other covariates that were not available in this retrospective dataset. Lastly, multiple comparisons and covariate-adjusted statistical analyses were not done and this can lead to an increase in the probability of type I errors. Based on this, it can be assumed that only correlational but not causal associations can be observed.

## 5. Conclusions

In our study, the diameters of cerebral veins were evaluated in a relatively large population aged 19–100 years of age. As the Fazekas score increased, the cerebral vein diameters increased. Additionally, it was determined that both the diameters of cerebral veins and the Fazekas score increased with age. The presence of hypertension, hyperlipidemia, diabetes mellitus, lacunar infarction and microhemorrhage was associated with an increase in cerebral vein diameters. Especially in middle-aged people, cerebral vein diameters can be evaluated to get an idea about the future course of WMH.

## Figures and Tables

**Table 1:** Descriptive statistical results of the participants.

Variables		Mean ± Standard Error Median (Minimum–Maximum)
Age, years		53.477 ± 0.678 55.00 (19–100)
ICV_right		1.504 ± 0.007 1.50 (1.20–2.00)
ICV_left		1.517 ± 0.016 1.50 (1.20–11.65)
SSS		7.082 ± 0.001 7.00 (5.10–9.90)
ASV_right		1.025 ± 0.001 1.020 (0.60–1.20)
ASV_left		1.021 ± 0.002 1.020 (0.04–1.10)
TSV_right		1.310 ± 0.006 1.30 (1.00–1.70)
TSV_left		1.296 ± 0.006 1.30 (0.70–2.00)
		n (%)
Hypertension	No	443 (67.1%)
	Yes	217 (32.9%)
Hyperlipidemia	No	525 (79.5%)
	Yes	135 (20.5%)
Diabetes Mellitus	No	526 (79.7%)
	Yes	134 (20.3%)
Lacunar Infarct	No	631 (95.6%)
	Yes	29 (4.4%)
Microhemorrhage	No	567 (85.9%)
	Yes	93 (14.1%)
Gender	Female	413 (62.6%)
	Male	247 (37.4%)
Fazekas	0	142 (21.5%)
	1	384 (58.2%)
	2	74 (11.2%)
	3	60 (9.1%)

ICV: internal cerebral vein, ASV: anterior septal vein, TSV: thalamostriate vein, SSS: superior sagittal sinuses.

**Table 2:** Comparison of participants’ data by gender.

		Gender			
		Female (n = 413)	Male (n = 247)	X^2^	p
Hypertension	No	261 (63.2%)	182 (73.7%)	7.704	0.006
	Yes	152 (36.8%)	65 (26.3%)		
Hyperlipidemia	No	323 (78.2%)	202 (81.8%)	1.213	0.271
	Yes	90 (21.8%)	45 (18.2%)		
Diabetes Mellitus	No	324 (78.5%)	202 (81.8%)	1.060	0.303
	Yes	89 (21.5%)	45 (18.2)		
Lacunar Infarct	No	398 (96.4%)	233 (94.3%)	1.525	0.217
	Yes	15 (3.6%)	14 (5.7%)		
Microhemorrhage	No	361 (87.4%)	206 (83.4%)	2.051	0.152
	Yes	52 (12.6%)	41 (16.6%)		
Fazekas	0	93 (22.5%)	49 (19.8%)	1.290	0.732
	1	241 (58.4%)	143 (57.9%)		
	2	44 (10.7%)	30 (12.1%)		
	3	35 (8.5%)	25 (10.1%)		
		Mean ± Standard error Median (minimum–maximum)		Z	p
Age, years		53.04 ± 0.83 55 (19–93)	54.20 ± 1.15 57.0 (19–100)	−1.005	0.315
ICV_right		1.490 ± 0.008 1.50 (1.20–2.00)	1.527 ± 0.012 1.50 (1.20–2.00)	−2.309	0.021
ICV_left		1.511 ± 0.026 1.45 (1.20–11.65)	1.527 ± 0.012 1.50 (1.20–1.95)	−2.532	0.011
SSS		6.995 ± 0.041 6.90 (5.10–9.90)	7.22 ± 0.054 7.10 (5.50–9.20)	−3.257	0.001
ASV_right		1.024 ± 0.001 1.02 (0.60–1.20)	1.026 ± 0.001 1.02 (0.97–1.10)	−0.643	0.520
ASV_left		1.021 ± 0.002 1.020 (0.60–1.20)	1.021 ± 0.004 1.02 (0.04–1.10)	−0.435	0.664
TSV_right		1.306 ± 0.007 1.30 (1.00–1.70)	1.316 ± 0.010 1.30 (1.00–1.65)	−0.693	0.489
TSV_left		1.290 ± 0.007 1.30 (0.80–1.70)	1.308 ± 0.010 1.30 (0.70–2.00)	−1.249	0.212

X^2^: Chi-Square test, Z: Mann–Whitney U test. ICV: internal cerebral vein, ASV: anterior septal vein, TSV: thalamostriate vein, SSS: superior sagittal sinuses.

**Table 3:** Relationship between participants’ cerebral vein diameters and the presence of hypertension, hyperlipidemia, diabetes mellitus, lacunar infarction and microhemorrhage.

	Hypertension			Hyperlipidemia			Diabetes Mellitus			Lacunar Infarct			Microhemorrhage		
	No	Yes	p	No	Yes	p	No	Yes	p	No	Yes	p	No	Yes	p
**ICV_right**	1.457 ± 0.008 1.40 (1.20–2.00)	1.599 ± 0.011 1.60 (1.20–2.00)	<0.001	1.488 ± 0.007 1.45 (1.20–2.00)	1.567 ± 0.016 1.60 (1.20–2.00)	<0.001	1.484 ± 0.007 1.42 (1.20–2.00)	1.581 ± 0.014 1.60 (1.20–1.90)	<0.001	1.495 ± 0.007 1.550 (1.20–2.00)	1.709 ± 0.026 1.72 (1.40–1.95)	<0.001	1.484 ± 0.007 1.45 (1.20–2.00)	1.627 ± 0.018 1.60 (1.20–2.00)	<0.001
**ICV_left**	1.457 ± 0.008 1.40 (1.20–1.95)	1.640 ± 0.047 1.60 (1.20–11.65)	<0.001	1.504 ± 0.020 1.45 (1.20–11.65)	1.568 ± 0.015 1.60 (1.20–1.90)	<0.001	1.499 ± 0.020 1.40 (1.20–11.65)	1.587 ± 0.014 1.60 (1.20–2.00)	<0.001	1.508 ± 0.017 1.45 (1.20–11.65)	1.704 ± 0.025 1.72 (1.30–1.90)	<0.001	1.499 ± 0.019 1.45 (1.20–11.65)	1.629 ± 0.017 1.65 (1.20–1.90)	<0.001
**ASV_right**	1.019 ± 0.001 1.01 (0.97–1.20)	1.037 ± 0.002 1.04 (0.60–1.10)	<0.001	1.023 ± 0.001 1.02 (0.60–1.20)	1.033 ± 0.011 1.03 (0.99–1.10)	<0.001	1.021 ± 0.001 1.02 (0.60–1.20)	1.041 ± 0.001 1.04 (1.00–1.09)	<0.001	1.024 ± 0.001 1.02 (0.60–1.20)	1.052 ± 0.004 1.06 (1.00–1.09)	<0.001	1.022 ± 0.001 1.02 (0.60–1.20)	1.041 ± 0.002 1.04 (1.00–1.09)	<0.001
**ASV_left**	1.015 ± 0.002 1.01 (0.04–1.09)	1.034 ± 0.003 1.04 (0.60–1.10)	<0.001	1.020 ± 0.002 1.02 (0.04–1.10)	1.025 ± 0.005 1.03 (0.60–1.10)	<0.001	1.019 ± 0.001 1.02 (0.60–1.10)	1.029 ± 0.008 1.04 (0.04–1.09)	<0.001	1.020 ± 0.002 1.02 (0.04–1.10)	1.053 ± 0.004 1.06 (1.00–1.09)	<0.001	1.020 ± 0.001 1.02 (0.60–1.10)	1.029 ± 0.011 1.04 (0.04–1.09)	<0.001
**TSV_right**	1.288 ± 0.007 1.25 (1.00–1.65)	1.355 ± 0.011 1.40 (1.00–1.70)	<0.001	1.302 ± 0.006 1.30 (1.00–1.70)	1.340 ± 0.014 1.30 (1.10–1.70)	0.017	1.296 ± 0.006 1.25 (1.00–1.70)	1.365 ± 0.013 1.40(1.00–1.70)	<0.001	1.304 ± 0.006 1.30 (1.00–1.70)	1.446 ± 0.028 1.50 (1.20–1.65)	<0.001	1.301 ± 0.006 1.30 (1.00–1.70)	1.362 ± 0.018 1.40 (1.00–1.70)	0.003
**TSV_left**	1.279 ± 0.007 1.20 (0.70–2.00)	1.332 ± 0.010 1.35 (0.90–1.65)	<0.001	1.290 ± 0.006 1.30 (0.80–2.00)	1.320 ± 0.015 1.30 (0.70–1.70)	0.047	1.287 ± 0.006 1.25 (0.80–2.00)	1.335 ± 0.015 1.40 (0.70–1.65)	<0.001	1.292 ± 0.006 1.30 (0.70–2.00)	1.393 ± 0.024 1.40 (1.10–1.65)	<0.001	1.292 ± 0.006 1.30 (0.70–2.00)	1.323 ± 0.016 1.30 (1.00–1.65)	0.073
**SSS**	7.142 ± 0.039 7.10 (5.10–9.20)	6.958 ± 0.060 6.90 (5.10–9.90)	0.004	7.150 ± 0.037 7.10 (5.10–9.90)	6.816 ± 0.067 6.70 (5.10–8.80)	<0.001	7.123 ± 0.036 7.10 (5.10–9.90)	6.921 ± 0.074 6.90 (5.50–9.90)	0.010	7.089 ± 0.033 7.00 (5.10–9.90)	6.917 ± 0.179 7.00 (5.40–8.70)	0.395	7.113 ± 0.035 7.00 (5.10–9.90)	6.891 ± 0.090 6.90 (5.40–9.00)	0.033

Mann–Whitney U test. ICV: internal cerebral vein, ASV: anterior septal vein, TSV: thalamostriate vein, SSS: superior sagittal sinuses.

**Table 4:** Relationship between participants’ cerebral vein diameters and Fazekas staging.

	Fazekas					
	0 (n = 142)	1 (n = 384)	2 (n = 74)	3 (n = 60)	Z	p
**ICV_right**	1.385 ± 0.011 1.40 (1.20–1.90)	1.478 ± 0.008 ^a^ 1.45 (1.20–2.00)	1.667 ± 0.015 ^a.b^ 1.69 (1.40–2.00)	1.752 ± 0.013 ^a.b.c^ 1.75 (1.50–2.00)	224.940	<0.001
**ICV_left**	1.386 ± 0.010 1.40 (1.20–1.90)	1.502 ± 0.027 ^a^ 1.45 (1.20–11.65)	1.660 ± 0.017 ^a.b^ 1.70 (1.30–2.00)	1.748 ± 0.010 ^a.b.c^ 1.75 (1.60–1.90)	214.257	<0.001
**ASV_right**	1.009 ± 0.001 1.00 (0.98–1.10)	1.021 ± 0.001 ^a^ 1.02 (0.06–1.20)	1.046 ± 0.002 ^a.b^ 1.05 (1.00–1.08)	1.063 ± 0.002 ^a.b.c^ 1.06 (1.00–1.10)	230.437	<0.001
**ASV_left**	1.005 ± 0.002 1.00 (0.70–1.10)	1.018 ± 0.003 ^a^ 1.02 (0.04–1.10)	1.041 ± 0.006 ^a.b^ 1.05 (0.60–1.08)	1.057 ± 0.008 ^a.b.c^ 1.07 (0.60–1.10)	229.130	<0.001
**TSV_right**	1.241 ± 0.011 1.20 (1.00–1.60)	1.289 ± 0.007 ^a^ 1.25 (1.00–1.65)	1.415 ± 0.016 ^a.b^ 1.40 (1.10–1.70)	1.480 ± 0.019 ^a.b.c^ 1.52 (1.10–1.70)	117.481	<0.001
**TSV_left**	1.239 ± 0.011 1.20 (0.80–1.50)	1.279 ± 0.007 ^a^ 1.25 (0.70–2.00)	1.390 ± 0.018 ^a.b^ 1.40 (1.00–1.70)	1.426 ± 0.022 ^a.b^ 1.50 (0.90–1.65)	86.807	<0.001
**SSS**	7.185 ± 0.069 7.15 (5.40–9.00)	7.099 ± 0.042 7.00 (5.10–9.90)	6.989 ± 0.112 ^a^ 6.85 (5.40–9.00)	6.840 ± 0.105 ^a.b^ 6.75 (5.50–8.60)	10.465	0.015
**Age**					X^2^	p
**Youth**	75 (52.8%)	85 (22.1%)	1 (1.4%)	0 (0%)	198.554	<0.001
**Middle age**	53 (37.3%)	153 (39.8%)	15 (20.3%)	4 (6.7%)		
**Elderly**	14 (9.9%)	146 (38.0%)	58 (78.4%)	56 (93.3%)		
**Hypertension**						
**No**	121 (85.2%)	271 (70.6%)	32 (43.2%)	19 (31.7%)	76.424	<0.001
**Yes**	21 (14.8%)	113 (29.4%)	42 (56.8%)	41 (68.3%)		
**Hyperlipidemia**						
**No**	124 (87.3%)	305 (79.4%)	56 (75.7%)	40 (66.7%)	12.081	0.007
**Yes**	18 (12.7%)	79 (20.6%)	18 (24.3%)	20 (33.3%)		
**Diabetes Mellitus**						
**No**	126 (88.7%)	315 (82.0%)	46 (62.2%)	39 (65.0%)	30.529	<0.001
**Yes**	16 (11.3%)	69 (18.0%)	48 (37.8%)	21(35.0%)		
**Lacunar infarct**						
**No**	142 (100%)	378 (98.4%)	68 (91.9%)	43 (71.7%)	98.138	<0.001
**Yes**	0 (0.0%)	6 (1.6%)	6 (8.1%)	17 (28.3%)		
**Microhemorrhage**						
**No**	134 (94.4%)	340 (88.5%)	60 (81.1%)	33 (55.0%)	59.366	<0.001
**Yes**	8 (5.6%)	44 (11.5%)	14 (18.9%)	27 (45.0%)		

X^2^: Chi-Square test, Z: Kruskal–Wallis test. ^a^ p < 0.05 compared to stage 0, ^b^ p < 0.05 compared to stage 1, ^c^ p < 0.05 compared to stage 2. ICV: internal cerebral vein, ASV: anterior septal vein, TSV: thalamostriate vein, SSS: superior sagittal sinuses.

**Table 5:** Relationship between participants’ cerebral vein diameters and age staging.

	Age				
	Youth (n = 161)	Middle-age (n = 225)	Elderly (n = 274)	X^2^	p
**ICV_right**	1.339 ± 0.006 1.34 (1.20–1.60)	1.434 ± 0.008 ^a^ 1.40 (1.20–1.80)	1.659 ± 0.008 ^a.b^ 1.66 (1.20–2.00)	365.297	<0.001
**ICV_left**	1.336 ± 0.006 1.32 (1.20–1.60)	1.439 ± 0.008 ^a^ 1.40 (1.20–1.80)	1.688 ± 0.037 ^a.b^ 1.67 (1.20–11.65)	343.297	<0.001
**ASV_right**	1.002 ± 0.0008 1.00 (0.97–1.05)	1.017 ± 0.001 ^a^ 1.02 (0.98–1.20)	1.045 ± 0.002 ^a.b^ 1.05 (0.60–1.10)	379.965	<0.001
**ASV_left**	1.003 ± 0.0008 1.00 (0.98–1.05)	1.011 ± 0.002 ^a^ 1.02 (0.60–1.10)	1.040 ± 0.004 ^a.b^ 1.05 (0.04–1.10)	367.851	<0.001
**TSV_right**	1.220 ± 0.010 1.20 (1.00–1.60)	1.278 ± 0.010 ^a^ 1.20 (1.00–1.60)	1.389 ± 0.009 ^a.b^ 1.40 (1.00–1.70)	128.235	<0.001
**TSV_left**	1.216 ± 0.010 1.20 (0.80–2.00)	1.273 ± 0.009 ^a^ 1.25 (1.00–1.60)	1.363 ± 0.009 ^a.b^ 1.40 (0.70–1.70)	111.278	<0.001
**SSS**	7.387 ± 0.064 7.40 (5.20–9.00)	7.063 ± 0.054 ^a^ 7.00 (5.10–9.20)	6.918 ± 0.519 ^a.b^ 6.80 (5.10–9.90)	35.272	<0.001

X^2^: Kruskal–Wallis test. ^a^ p < 0.05 compared to the youth group, ^b^ p < 0.05 compared to the middle-aged group. ICV: internal cerebral vein, ASV: anterior septal vein, TSV: thalamostriate vein, SSS: superior sagittal sinuses.

**Table 6:** Correlation of participants’ cerebral vein diameters with age.

		ICV_Right	ICV_Left	SSS	ASV_Right	ASV_Left	TSV_Right	TSV_Left
**Age**	r	0.748	0.735	−0.230	0.786	0.735	0.466	0.434
	p	<0.001	<0.001	<0.001	<0.001	<0.001	<0.001	<0.001
**Adjusted Age**	r	0.648	0.295	−0.160	0.516	0.267	0.391	0.365
	p	<0.001	<0.001	<0.001	<0.001	<0.001	<0.001	<0.001

^r^ Spearman correlation coefficient. ICV: internal cerebral vein, ASV: anterior septal vein, TSV: thalamostriate vein, SSS: superior sagittal sinuses.

**Table 7:** Kolmogorov–Smirnov test for normality.

Variable	K–S Z	p Value	Distribution
**Age**	0.214	<0.001	Non-normal
**Fazekas score**	0.367	<0.001	Non-normal
**ICV-Right**	0.198	<0.001	Non-normal
**ICV-Left**	0.203	<0.001	Non-normal
**ASV-Right**	0.176	<0.001	Non-normal
**ASV-Left**	0.181	<0.001	Non-normal
**TSV-Right**	0.164	<0.001	Non-normal
**TSV-Left**	0.169	<0.001	Non-normal
**SSS**	0.221	<0.001	Non-normal

**Table 8:** Multiple-comparison-corrected correlations.

Variable	Spearman r	Raw p	Adjusted p	Significance
**ICV-Right**	0.71	<0.001	<0.001	Significant
**ICV-Left**	0.69	<0.001	<0.001	Significant
**ASV-Right**	0.67	<0.001	<0.001	Significant
**ASV-Left**	0.65	<0.001	<0.001	Significant
**TSV-Right**	0.48	<0.001	<0.001	Significant
**TSV-Left**	0.46	<0.001	<0.001	Significant
**SSS**	−0.21	0.009	0.063	Not significant

**Table 9:** Non-parametric ANCOVA (Quade’s test).

Dependent Variable	F Value	p Value	Partial η^2^
**ICV-Right**	32.6	<0.001	0.18
**ICV-Left**	30.9	<0.001	0.17
**ASV-Right**	27.4	<0.001	0.15
**ASV-Left**	25.8	<0.001	0.14
**TSV-Right**	14.3	<0.001	0.08
**TSV-Left**	13.1	<0.001	0.07
**SSS**	2.4	0.091	0.01

**Table 10:** Random Forest variable importance.

Predictor	Mean Decrease Gini	Relative Importance (%)
**Age**	41.2	100
**Hypertension**	19.4	47
**Diabetes mellitus**	15.7	38
**Hyperlipidemia**	12.3	30
**ICV-Right**	10.9	26
**ICV-Left**	10.5	25
**ASV-Right**	8.6	21
**ASV-Left**	8.1	20
**TSV-Right**	6.9	17
**TSV-Left**	6.4	16

**Table 11:** SHAP summary statistics for age.

Age Range (Years)	Mean SHAP Value	Direction
**<40**	−0.62	Decreases WMH severity
**40–60**	0.18	Mild increase
**61–80**	0.74	Moderate increase
**>80**	1.31	Strong increase

## Data Availability

The datasets generated during and/or analyzed during the current study are available from the corresponding author on reasonable request.
